# General and Genomic DNA-Binding Specificity for the *Thermus thermophilus* HB8 Transcription Factor TTHB023

**DOI:** 10.3390/biom10010094

**Published:** 2020-01-06

**Authors:** James Shell Cox, Michael W. Van Dyke

**Affiliations:** 1 Department of Chemistry and Biochemistry, Kennesaw State University, Kennesaw, GA 30144, USA; 2 Medical College of Georgia, Augusta University, Augusta, GA 30912, USA

**Keywords:** DNA-binding specificity, REPSA, *Thermus thermophilus* HB8, TTHB023, transcriptional regulation

## Abstract

Transcription factors are proteins that recognize specific DNA sequences and affect local transcriptional processes. They are the primary means by which all organisms control specific gene expression. Understanding which DNA sequences a particular transcription factor recognizes provides important clues into the set of genes that they regulate and, through this, their potential biological functions. Insights may be gained through homology searches and genetic means. However, these approaches can be misleading, especially when comparing distantly related organisms or in cases of complicated transcriptional regulation. In this work, we used a biochemistry-based approach to determine the spectrum of DNA sequences specifically bound by the *Thermus thermophilus* HB8 TetR-family transcription factor TTHB023. The consensus sequence 5′–(a/c)Y(g/t)A(A/C)YGryCR(g/t)T(c/a)R(g/t)–3′ was found to have a nanomolar binding affinity with TTHB023. Analyzing the *T. thermophilus* HB8 genome, several TTHB023 consensus binding sites were mapped to the promoters of genes involved in fatty acid biosynthesis. Notably, some of these were not identified previously through genetic approaches, ostensibly given their potential co-regulation by the *Thermus thermophilus* HB8 TetR-family transcriptional repressor TTHA0167. Our investigation provides additional evidence supporting the usefulness of a biochemistry-based approach for characterizing putative transcription factors, especially in the case of cooperative regulation.

## 1. Introduction

The process of transcription is the primary means by which all organisms control their specific gene expression. This is achieved through the binding of sets of proteins known as transcription factors to specific DNA sequences often located within the promoter regions of regulated genes. Identifying those genes can provide valuable information regarding a transcription factor and its biological role in an organism’s physiology.

Advances in DNA sequencing techniques have allowed the determination of genomic sequences for over 370 thousand organisms [1]. Within these, dozens to hundreds of potential transcription factors can be identified using bioinformatics tools. However, it is beyond our present ability to use this sequence information to accurately predict those DNA sequences recognized by a presumed transcription factor and the target genes it regulates. Simple inferences, e.g., assuming transcription factor autoregulation of its own and opposing operons and recognition of palindromic sequences by homodimeric transcription factors, can provide useful leads as to the specific DNA sequences recognized by these proteins and the types of genes that they regulate [2]. However, a more thorough understanding typically requires experimentally derived data.

In organisms for which we have tractable genetic tools, an approach involving transcription factor gene disruption, measured changes in global gene expression, and bioinformatic homology studies can be used to obtain information ranging from defining regulatory regions to potential biological roles for relatively uncharacterized transcription factors. Such an approach has proven highly effective in the model organism *Escherichia coli*, where information regarding its transcription factors, their consensus binding sequences, and the genes that they regulate, has been obtained [3].

Unfortunately, genetic tools are not always practical in all organisms for which genomic information is available. Thus, there exists a need for alternative approaches for characterizing transcription factors in these organisms. We have developed an alternative, biochemistry-based approach, using the selection method Restriction Endonuclease Protection, Selection, and Amplification (REPSA), massively parallel sequencing, and bioinformatics to determine a consensus binding sequence and thereby identify possible genes regulated by these transcription factors. We have successfully used this approach to investigate three putative TetR-family transcription factors, TTHA0167, TTHA0101, and TTHA0973, in the extreme thermophile *Thermus thermophilus* HB8 [4,5,6]. Here, we describe our investigations into the DNA-binding specificity and genomic targets of TTHB023, a putative TetR-related transcriptional repressor protein in *T. thermophilus* HB8. This study further improves the use of REPSA in identifying consensus binding sequences and provides further insights into potential transcription factor functions in an important model organism [7].

## 2. Results

### 2.1. In Vitro Selection of DNAs That Bind TTHB023

**Figure 1:**
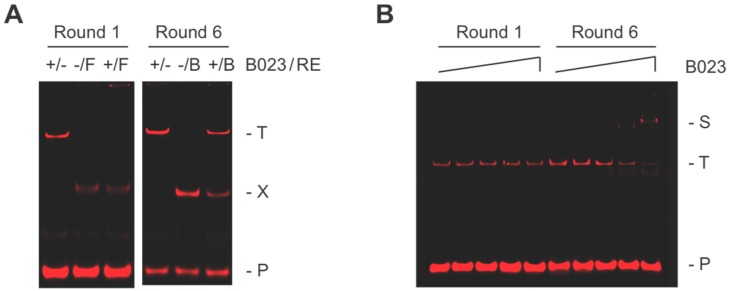
Selection and validation of TTHB023-binding DNA sequences. (**A**) Shown are IR fluorescence images of cleavage protection assays from Round 1 and Round 6 of Restriction Endonuclease Protection, Selection and Amplification (REPSA) selection with 17 nM TTHB023 protein. The presence (+) or absence (−) of TTHB023 and IISREs FokI (F) or BpmI (B) is indicated above each lane. Locations of intact (T) and cleaved (X) ST2R14 selection template and the IRD7_ST2R primer (P) are indicated at right of figure. (**B**) Shown are IR fluorescence images of Electrophoretic Mobility Shift Assays (EMSAs) made with DNA from REPSA Round 1 (left lanes) and Round 6 (right lanes) incubated with increasing TTHB023 concentrations (from left to right: 0, 0.83, 8.3, 83, or 830 nM TTHB023). Electrophoretic mobility of a single TTHB023–DNA complex (S) as well as the uncomplexed template (T) and primer (P) are indicated at figure right.

The selection method REPSA, which relies on sequence-specific DNA binding by a ligand interfering with cleavage by a type IIS restriction endonuclease (IISRE), was used to screen billions of sequences to identify those preferentially bound by the TTHB023 protein. The selection template and experimental methods used were as previously described [4]. Twenty-five billion ST2R24 molecules were used to initiate REPSA, providing a greater than 10-fold excess over the number of 16-bp sequences possible (~2.15 billion). Three rounds of REPSA were performed with the IISRE FokI, followed by three rounds with the IISRE BpmI. As shown in Figure 1A (left panel), Round 1 REPSA-selected DNAs were not resistant to FokI cleavage in the presence of TTHB023. However, by Round 6 (right panel), most DNAs were cleavage resistant when TTHB023 was present. The selection of TTHB023-binding sequences was further demonstrated by EMSA. A slower mobility species was only observed with Round 6 DNAs incubated with high concentrations of TTHB023 and not with Round 1 DNAs (Figure 1B), indicating that Round 6 DNAs contained specific TTHB023 binding sites.

**Figure 2:**
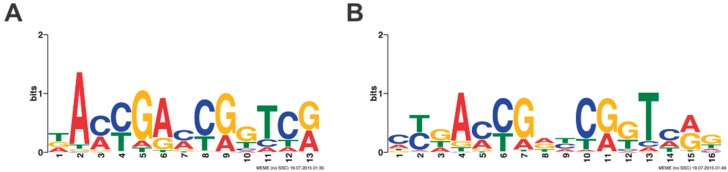
TTHB023 consensus sequences. Multiple Em for Motif Elicitation (MEME) software was used with 1000 Round 6 DNA sequences and either (**A**) no filters or (**B**) with a palindromic filter.

A fusion PCR amplicon library was generated from Round 6 REPSA-selected DNAs and sequenced using an Ion Personal Genome Machine. Sequencing yielded 4,713,006 total bases, 3,369,036 ≥ Q20, and 99,077 reads of 48 bp mean length (Data S1). Using our Sequencing1.java program, we obtained 7453 sequences (Data S2) that were subsequently analyzed using Multiple Em for Motif Elicitation (MEME) software [8]. Sequence logos obtained included a nonpalindromic 13-mer (Figure 2A) and a 16-mer palindromic motif (Figure 2B). Notably, these motifs were found in nearly all sequences (palindromic: 992/1000, nonpalindromic: 1000/1000), with E-values of 7.2 × 10^−1942^ and 1.1 × 10^−1650^, respectively. Given the extremely high statistical significance of these E-values and that TTHB023, as a TetR family protein, would likely bind a palindromic site as a homodimer, we would expect the palindromic sequence to be the consensus recognition sequence for TTHB023.

**Figure 3:**
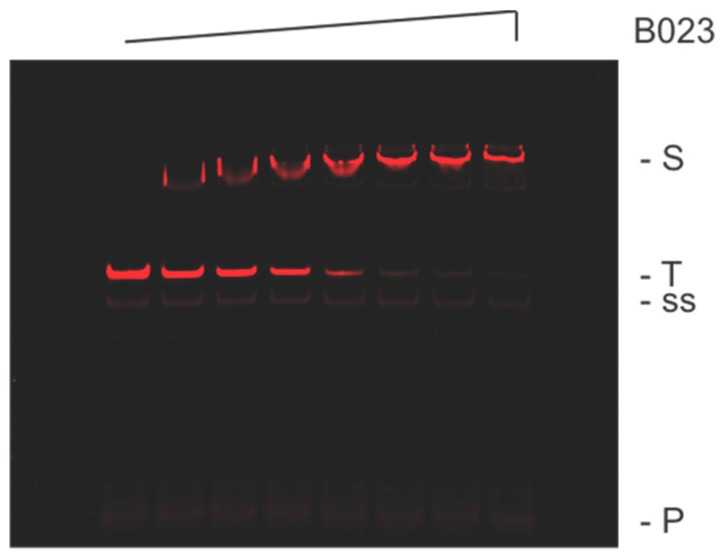
EMSA analysis of TTHB023-binding to its consensus sequence. Shown are IR fluorescence images of TTHB023 palindromic DNA incubated with 0, 13, 26, 52, 104, 208, 415, or 830 nM TTHB023 protein. (S) Protein–DNA complex, (T) uncomplexed DNA, (ss) single-stranded DNA, and (P) IRD7_ST2R primer.

We next used quantitative EMSA to validate our TTHB023 consensus sequence. We observed a TTHB023 concentration-dependent slower mobility species consistent with specific binding (Figure 3). An apparent dissociation constant, K_D_ = 65 nM, could be determined from these data. Note that TTHB023 appeared to be dissociating from its DNA complex under our electrophoretic conditions, as evident from the smearing of the shifted species at lower TTHB023 concentrations. Thus, dissociation constants for this complex may be underestimated using quantitative EMSA under these conditions.

**Figure 4:**
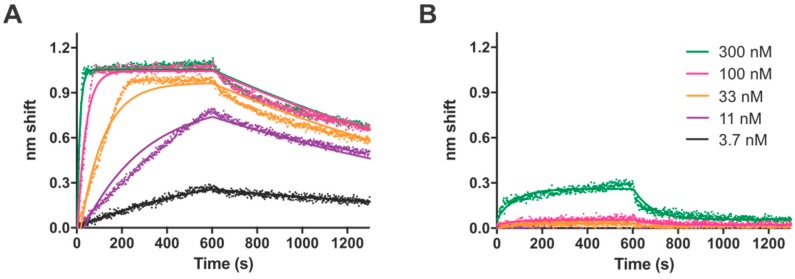
Biolayer Interferometry (BLI) analysis of TTHB023-DNA binding. Shown are raw traces (dots) and best-fit lines of TTHB023 binding to (**A**) ST2_B023_R6_wt consensus DNA or (**B**) REPSAis control DNA. TTHB023 concentrations investigated include 300 nM (green), 100 nM (magenta), 33 nM (gold), 11 nM (violet), and 3.7 nM (black).

To avoid problems with the electrophoretic stability of TTHB023–DNA complexes, we used the solution-based binding assay biolayer interferometry (BLI) to characterize the binding kinetics of TTHB023 to different DNAs (Table S1). Graphical representations of BLI experiments with the TTHB023 consensus sequence or a control DNA are shown in Figure 4. The consensus sequence demonstrated appreciable interference changes with increasing TTHB023 concentration, whereas the control DNA only demonstrated slight effects at the highest concentration investigated, consistent with nonspecific binding. For each DNA, single-state association and dissociation kinetics and an apparent dissociation constant were calculated using GraphPad Prism software (Table 1). In each case, the global goodness-of-fit value was quite good (R^2^ ≥ 0.9399), suggesting the kinetic values were accurate for TTHB023-DNA binding. Data from these BLI experiments with different consensus sequence mutants were generally consistent with the significance of individual nucleotides seen in our sequence logos. This further points to the utility of REPSA in identifying high-affinity protein-DNA binding sequences.

### 2.2. T. thermophilus HB8 Genomic TTHB023-Binding Site Identification

**Figure 5:**
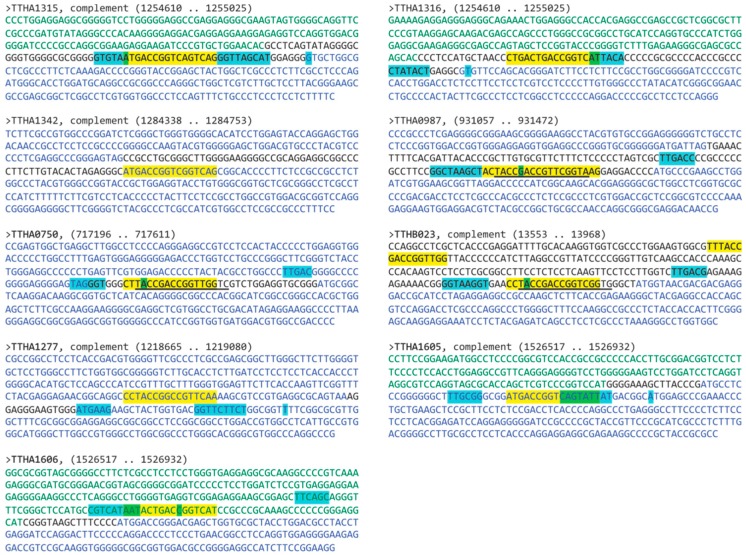
Bioinformatic identification of *T. thermophilus* HB8 promoters bound by TTHB023. Shown are sequences +/− 200 bp of the Find Individual Motif Occurrences (FIMO)-identified TTHB023-binding sequence (see Table 2). Text colors indicate open reading frames with the same orientation as the target gene (blue nucleotides), intergenic sequences (blue nucleotides), and opposite-oriented open reading frames (green nucleotides). Highlighting indicates promoter elements (cyan highlighting), TTHB023-binding sites (yellow highlighting), and overlapping TTHB023-binding/core promoter elements (green highlighting).

To identify potential TTHB023 binding sites within the *T. thermophilus* HB8 genome, the Find Individual Motif Occurrences (FIMO) program [9] was used to probe the *Thermus thermophilus* HB8 uid13202 210 database with the 16-mer palindromic TTHB023 position-weight matrix. Ninety-six motif occurrences with a *p*-value of less than 0.0001 were obtained. Those occurrences whose significance (*p*-values) were ≤ 5.10 × 10^−6^ are shown in Table 2. These were mapped to the *T. thermophilus* HB8 genome and sequences ±200 bp of these sites were scanned for potential bacterial core promoter elements using Softberry BPROM software [10]. These data are shown in Figure 5. Most of the highest significance TTHB023 binding sites were located in identifiable promoter regions, e.g., *TTHA1315*, *TTHA1316*, *TTHA0987*, *TTHA0750*, *TTHB023*, *TTHA1605*, and *TTHA1606*. A minority of high significance TTHB023 sites were located within postulated genes or without identifiable promoters. These are less likely to be involved in TTHB023-mediated gene regulation and were not subject to further study. Using available databases such as ProOpDB and BioCyc [11,12], most TTHB023-binding sites (e.g., *TTHA1315/16, TTHA0987*, *TTHB023*, and *TTHA1605/06*) could be mapped to locations upstream of genes at the beginning of operons or single transcriptional units, supporting their possible involvement in transcriptional regulation (Table 2). One exception, *TTHA0750*, is positioned near the end of a postulated operon. However, the presence of a potential core promoter overlapping the TTHB023 binding site prompted us to keep it in consideration.

### 2.3. TTHB023-Regulated Gene Validation

To validate candidate genes for TTHB023 regulation, we used BLI to determine TTHB023 binding kinetics to each promoter binding site. TTHB023 binding sites upstream of genes *TTHA0987*, *TTHA0750*, and *TTHB023* demonstrated high-affinity binding (K_D_ < 1 nM), while *TTHA1315/16* and the *TTHA0987*(−14) site showed intermediate binding affinities (5 to 7 nM) (Table 3). Very weak binding affinity (836 nM) was observed for the TTHB023 binding site found upstream of the *TTHA1605/06* bidirectional promoters, which makes it a less likely site for TTHB023 occupancy in vivo. We consider the high and intermediate TTHB023-binding sites to be the most relevant physiologically, having the best potential of regulating operon genes downstream.

To better ascertain whether TTHB023 may be involved in the regulation of these genes, we used publicly available National Center for Biotechnology Information Gene Expression Omnibus (NCBI GEO) expression profile data from wild-type and TTHB023-deficient strains of *T. thermophilus* HB8 [13]. GEO2R analysis of expression profiles was used to rank tags according to their adjusted *p*-values for differential expression in the absence of TTHB023 (Table S2). GEO2R data for those genes that have identified TTHB023-binding sites in their promoters are shown in Table 4. Several of these genes showed a significant change in expression, with both upregulation (positive logFC values) and downregulation (negative logFC values) being observed. Three of our identified TTHB023-responsive genes, *TTHB023*, *TTHA0750*, and *TTHA0987*, were in the top twenty GEO2R candidates. Both *TTHA0750* and *TTHA0987* exhibited moderate (3.6- ~ 3.9-fold) upregulation in TTHB023-deficient strains, whereas, *TTHB023* was significantly (365-fold) downregulated in these strains. Such data are consistent with the effective disruption of *TTHB023* genes in polyploid *T. thermophilus* HB8 and *TTHB023* serving as a transcriptional repressor protein for *TTHA0750* and *TTHA0987* [14].

### 2.4. T. thermophilus HB8 TTHB023 Postulated Biological Roles

Given TTHB023 binding site locations within the *T. thermophilus* HB8 genome and TTHB023 binding affinity to these sites, coupled with bioinformatic identification of gene promoters and defined operons, it becomes possible to postulate those genes that TTHB023 most likely regulates. These are shown in Table 5. Members included genes in the *TTHB023* operon and several single transcriptional units. Using available bioinformatics databases (e.g., KEGG, UniProtKB [16,17]), we find that many TTHB023-regulated genes encode enzymes that could be involved in fatty acid degradation, although alternative metabolic pathways (e.g., leucine, isoleucine, and valine degradation) may also be affected. Thus, it is reasonable to assume that TTHB023 regulates fatty acid metabolism and may be responsive to a metabolite generated through that process.

## 3. Discussion

In this paper, we report our biochemistry-based investigation on the *T. thermophilus* HB8 transcription factor TTHB023. Using REPSA selection, massively parallel sequencing, and MEME motif elicitation, we found that TTHB023 preferentially binds the consensus sequence, 5′–(a/c)Y(g/t)A(A/C)YGryCR(g/t)T(c/a)R(g/t)–3′. In comparison, Agari et al. functionally identified TTHB023-regulated genes based on their magnitude change in expression when comparing mRNAs isolated from wild-type and Δ*pfmR* strains [18]. Comparing the nucleotide sequences upstream of 30 candidate genes, they found three, *TTHA0750*, *TTHA0987*, and *TTHB023*, containing similar pseudopalindromic sequences in their upstream regions. These sequences allowed them to derive a predicted consensus TTHB023-binding site, 5′–TACCGACCGNTNGGTN–3′. Notably, there is a significant degree of overlap between our consensus sequence and that derived by Agari et al. (see underlining, above), suggesting that they both encompass bona fide TTHB023 recognition sequences. However, given the considerable difference in sample size (992 versus 3) and statistical significance (E-values 7.2 × 10^−1942^ versus 9.3 × 10^−6^) between the two studies, the consensus we report is likely to be a more accurate representation of the spectrum of DNA-binding sites preferentially recognized by TTHB023.

Having defined a consensus recognition sequence, we sought to characterize the TTHB023-DNA interaction biophysically through the use of BLI. DNA probes containing either the consensus sequences or selected point mutations were analyzed, to better ascertain the importance of each nucleotide within the consensus. We found a dissociation constant of 2 nM for the consensus sequence and a range of 4.5 to 279 nM for the different point mutants. Notably, these dissociation constants closely mirrored the statistical significance of each nucleotide position from the MEME-derived position-weight matrix, as represented in the TTHB023 palindromic sequence logo. Thus, these data validate the sequences obtained by our REPSA-based approach as being those DNAs with high-affinity TTHB023 binding sites. Agari et al. performed a much more limited biophysical analysis of TTHB023-DNA binding, choosing to only investigate TTHB023 binding to its own gene’s upstream region using the technique surface plasmon resonance [18]. Most important, they found similar *k*_on_ (5.6 ± 1.1 × 10^5^ M^−1^·s^−1^), *k*_off_ (4.3 ± 0.3 × 10^−3^ s^−1^), and K_D_ (7.9 ± 1.4 × 10^−9^ M) values to what we observed, giving additional credence that the high-affinity binding we both found may be an accurate representation of TTHB023-binding parameters under physiological conditions.

In our biochemistry-based approach, we use a consensus sequence and bioinformatics to identify potential protein binding sites within an organism’s genome. With TTHB023, we initially obtained 96 motif occurrences, which were then reduced to nine based on their homology with the consensus sequence (*p*-value ≤ 5.10 × 10^−6^) and location within an identifiable promoter region. These nine included two bi-directional promoter regions (*TTHA1315*/*16*, *TTHA1605*/*06*) and two promoters (*TTHA0987, TTHB023*) containing two TTHB023 binding sites each. Interestingly, four of these sites overlapped with those identified by Agari et al. (Figure 5, indicated by underlining) [18]. Our sites were validated using BLI, which found that all but the *TTHA1605/06* shared site exhibited high-affinity TTHB023 binding (K_D_ from 0.3 to 7.4 nM) comparable to that observed with the consensus sequence. These data strongly suggest that TTHB023 could bind and regulate these promoters in vivo. Special note should be made regarding the location of TTHB023 binding within the promoter region. In many cases (e.g., *TTHA0987, TTHA0750, TTHB023*), the TTHB023-binding sites encompassed their mapped start sites of transcription (+1 site). Such would be expected for a transcriptional repressor that hinders the process of promoter-bound RNA polymerase transitioning to a productive transcriptional state rather than blocking RNA polymerase-promoter access. Thus, TTHB023 may behave more like the QacR regulator in *Staphylococcus aureus* than a typical TetR-family transcriptional repressor [19,20].

Given that TTHB023 binding sites typically overlapped with core promoter elements (−35 and −10 boxes, +1 site) and that TTHB023 is structurally related to the TetR transcriptional repressor protein, it is quite likely that TTHB023 functions as a transcriptional repressor. Agari et al. used in vitro transcription assays to validate TTHB023 function on the promoters they identified [18]. In accordance with a role as a transcriptional repressor, they found decreasing transcription with increasing TTHB023 concentration. However, rather high (100 to 500 nM) TTHB023 concentrations were necessary in order to observe appreciable inhibition on most promoters, and similar transcription suppression was observed on a promoter (*TTHA0973*) not thought to have a TTHB023 binding site. These findings demonstrate that while validation of transcription factor function on a promoter is important, methods like in vitro transcription have their limitations.

We used available microarray data from wild-type and Δ*pfmR* strains of *T. thermophilus* HB8 to identify those genes whose expression is most affected by the loss of TTHB023 under normal growth conditions [13]. We found three of the genes we identified, *TTHB023*, *TTHA0750*, and *TTHA0987*, to be among the top 20 genes that exhibited the greatest change in transcript levels between wild-type and deletion strains. *TTHB023* exhibited a significant (365-fold) decrease in transcripts, as expected, given that this was the gene disrupted in the Δ*pfmR* strains. *TTHA0750* and *TTHA0987* both exhibited moderate (3.6 to 3.9-fold) increases in expression, consistent with TTHB023 serving as a transcriptional repressor on their promoters. Further analysis of downstream genes being affected by *TTHB023* depletion found others in the operon *TTHB023*–*TTHB014* that were also upregulated, albeit at only moderate levels (1.9 to 3.6-fold). Such is not entirely unexpected, given that prokaryotic transcriptional repressors often suppress the expression of their operons, as part of a negative-feedback regulation loop [2,21]. Examining the reported roles of the *TTHB023*–*TTHB014* gene products, one finds that many are enzymes potentially involved in fatty acid metabolism. Agari et al. took this information one further, stating that these enzymes may be involved in phenylacetic acid degradation and fatty acid degradation and biosynthesis [18]. Thus, they refer to TTHB023 as the regulator of phenylacetic acid and fatty acid metabolism, or PfmR.

In our analysis of the *T. thermophilus* HB8 genome, we found two gene pairs, *TTHA1315/16* and *TTHA1606/06*, that contained potential TTHB023 binding sites within their promoter regions but were not identified by Agari et al. [18]. Such is understandable as their expression was not appreciably affected when *TTHB023* was deleted. We do not believe that *TTHA1606/06* is an actual target of TTHB023, given its relatively weak binding affinity. However, *TTHA1315/16* does possess an intermediate affinity TTHB023 binding affinity site, making these genes potential targets. One complication with standard genetic approaches for identifying targets of transcription factors is that not all genes have simple regulation programs, i.e., are regulated by a single transcription factor. Many are combinatorially regulated, involving both transcriptional repressors and activators [21]. Thus, depletion of a single transcription factor may have, at best, only moderate effects. In the case of *TTHA1315/16*, we believe multiple transcriptional repressors may be involved in their regulation. In fact, we had previously shown that these genes are likely targets of another *T. thermophilus* HB8 TetR-family transcriptional repressor, TTHA0167, otherwise known as SbtR [4,22]. We found that the consensus sequence for TTHA0167 is 5′–TGACYrnnyRGTCA–3′, which can be accommodated by the central 14 nucleotides of the TTHB023 consensus sequence in many cases. We have performed BLI measurements with TTHA0167 on the TTHB023 binding sites found in the *TTHA1315/16* and *TTHA1606/06* promoter regions and found evidence for high-affinity binding, with K_D_ values in the 1–2 nM range (Table 6). These data strongly suggest that the *TTHA1315* and *TTHA1316* genes may be regulated by both the TTHA0167 and TTHB023 transcriptional repressors, requiring multiple effectors to be present to permit their maximal expression in vivo. This co-ordinate regulation may also help explain why previous investigators did not report *TTHA1315* and *TTHA1316* being regulated by TTHA0167 in vivo, even though they well recognized TTHA0167 binding to these gene promoters in genomic selection experiments [22]. We were able to confirm this using publicly available microarray data and GEO2R, finding that *TTHA1315* and *TTHA1316* are not among the top 250 genes whose expression is affected by *TTHA0167* deletion (Table S3). Taken together, these examples point to an inherent strength of a biochemistry-focused approach for transcription factor discovery, especially in cases involving complicated regulatory schemes.

## 4. Materials and Methods

### 4.1. Oligonucleotides

Oligonucleotides (Table S1) were purchased from Integrated DNA Technologies. REPSA and sequencing libraries were prepared as previously described [4]. The initial REPSA library was prepared by PCR amplification of ST2R24 template with primers ST2L and IRD7_ST2R for six cycles, thereby ensuring maximal double-stranded DNA product with properly annealed randomized cassette regions. Subsequent REPSA round libraries were PCR amplified for 6, 9, and 12 cycles, thereby allowing the identification of optimally amplified, properly annealed DNA as above. Sequencing libraries were prepared by a two-step fusion PCR process, using primers A_BC02_ST2R and trP1_ST2L as the initial set and A_uni and trP1_uni as the second set. EMSA and BLI probes were made by PCR [23], using primer sets ST2L and IRD7_ST2R or ST2L and Bio_ST2R, respectively.

### 4.2. Protein Expression and Purification

The TTHB023 protein was expressed and purified from *E. coli* BL21(DE3):pET-ttPfmR bacteria following procedures described previously [4]. Bacterial cultures (50 mL) in LB medium + 100 µg/mL ampicillin were expanded to mid-log phase (A_590_ = 0.4) before inducing with 1 mM isopropyl β-D-1-thiogalactopyranoside (IPTG). Cultures were maintained for an additional 4 h before harvesting by centrifugation. Bacterial extracts were prepared by resuspending the bacterial pellet in 0.5 mL cold 40 mM Tris-Cl (pH 7.7) + 200 mM NaCl followed by sonication (3 W, 20 s bursts, ×5) and subsequent clarification by centrifugation (21,000× *g*, 10 min, 4 °C). Purification was achieved by heat-treating the soluble fraction at 70 °C for 10 min, followed by centrifugation as above. Analysis by SDS-PAGE and P200 ScreenTape assay (Figure S1) indicated that the TTHB023 preparation used in this study was more than 75% pure.

### 4.3. Transcription Factor Consensus Sequence Determination

REPSA selections were performed following procedures described previously [4], except that TTHB023 was used in the selections and the IISRE FokI was used in Rounds 1–3, while BpmI was used in Rounds 4–6. REPSA reactions (20 µL) consisted of 2 ng (42 fmoles) DNA from the previous round of selection plus 17 nM TTHB023 protein, as indicated, in 1 × New England Biolabs CutSmart buffer. Reactions were incubated for 20 min at 55 °C to permit specific binding. Following 5 min cooling to 37 °C, 0.2 U IISRE was added, and incubations continued for an additional 5 min before stopping on ice. DNA cleavage was analyzed by native PAGE and IR fluorescence imaging, as described [24]. Comparisons were made between reactions without IISRE, without TTHB023, and with both being present (see Figure 1, Lanes 1, 2, and 3, respectively) to determine if ligand-dependent, cleavage resistance were present.

Once TTHB023-dependent IISRE cleavage resistance was observed (Round 6), a sequencing library was generated as described above. These were then massively parallel sequenced using a Thermo Fisher Scientific semiconductor sequencer as previously described [4]. Raw sequence data in fastq format is provided in Supplementary Materials Data S1. These were then processed using our Sequencing1.java program to identify sequences suitable for further analysis and are provided in Data S2.

To determine a TTHB023 consensus DNA binding sequence, the Sequencing1.java-refined DNA sequences were subjected to Multiple Em for Motif Elicitation (MEME) analyses [13]. MEME discovers novel, ungapped, recurring fixed-length patterns from supplied sequences using statistical modeling techniques to automatically determine the best sequence width, number of occurrences, and description for each motif. It represents these motifs as position-dependent, letter-probability matrices that describe the probability of a particular nucleotide being present at any location within the motif. These position-weight matrices may then be visually represented as sequence logos, from which a consensus sequence may be derived using the most likely nucleotides at each position. Meme Analyses were performed with and without palindromic sequence filtering, with the former being used to generate a TTHB023 consensus sequence, given the likelihood that this TetR-family protein binds DNA as a homodimer and recognizes a palindromic sequence.

### 4.4. Protein-DNA Binding Assays

Electrophoretic mobility shift assays (EMSAs) with both Round 6 pool and TTHB023 consensus DNA were performed as previously described [4,5], with a detailed protocol being available [24]. Binding reactions (5 µL) consisted of 1 ng (21 fmoles) DNA and indicted concentration of TTHB023 in 1 × NEB CutSmart buffer. After incubation (20 min, 55 °C), 2 µL 20% glucose + 0.9% Orange G was added and samples analyzed by 10% native PAGE, as described [4,24]. Protein–DNA complexes and free DNA were identified and quantitated using IR fluorescence imaging. Apparent dissociation constants were then determined using data from the binding midpoint (approx. 50:50 complex:free DNA) and a standard binding equilibrium equation, as previously [4,24].

Biolayer interferometry (BLI), which measures real-time changes in interference pattern wrought by the binding of macromolecules to a biosensor tip, was performed using a Molecular Devices FortéBio Octet QK system as previously described [4]. Biotinylated probes containing either the consensus sequence, point mutations thereof, or binding sites identified in gene promoters were synthesized by standard PCR using the Bio-ST2R and ST2L primers and templates listed in Table S1. Solutions containing 1.4 nM biotinylated DNA in BLI buffer (20 mM Tris-Cl [pH 7.7], 100 mM NaCl, 1 mM EDTA, 0.05% Tween 20) were initially incubated with instrument-affixed Streptavidin Biosensors for 15 min at 37 °C to afford complete binding. Following a wash and baseline step, these biosensors were then incubated with five TTHB023 concentrations (3.7, 11, 33, 100, 300 nM) in BLI buffer for 5 min to permit protein-DNA association, followed by incubation in BLI buffer alone to allow dissociation. Interference pattern change magnitudes obtained every 1.6 s during the association and dissociation steps were analyzed by GraphPad Prism software using their single-state Association then Dissociation equation. Nonlinear fit results, including kinetic on and off rates (*k*_on_, *k*_off_) as well as calculated equilibrium binding constants (K_D_), were determined. Raw data and best-fit curves were shown graphically.

### 4.5. Bioinformatic Determination of Candidate Regulated Genes

To identify TTHB023-binding sites within the *T. thermophilus* genome, the 16-bp palindromic position weight matrix was used as the input to a Find Individual Motif Occurrences (FIMO) analysis (http://meme-suite.org/tools/fimo) [9]. Stringency was limited to include matches having *p*-values < 5.10 × 10^−6^. Sequences ±200 bp from the TTHB023 binding site obtained from the KEGG database (https://www.genome.jp/kegg-bin/show_organism?org=T00220) were analyzed by the Softberry BPROM (http://www.softberry.com) program to identify potential bacterial core promoter elements [10,16]. Operons were identified using the ProOpDB database at the Universidad Nacional Autónoma de México (http://biocomputo2.ibt.unam.mx/OperonPredictor/) and data from BioCyc (http://biocyc.org) [11,12]. Putative biological functions of TTHB023-regulated genes were obtained using available *T. thermophilus* HB8 databases at KEGG and UniProtKB (https://www.uniprot.org [300852]) [16,17].

To ascertain whether TTHB023 was a regulator of target genes, publicly available microarray data for gene expression profiles in wild-type and TTHB023-deficient *T. thermophilus HB8* were obtained from the National Center for Biotechnology Information Gene Expression Omnibus (NCBI GEO) website (https://www.ncbi.nlm.nih.gov/geo/) [13]. SuperSeries GSE36912, specifically samples GSM904786-8, obtained from wild-type *T. thermophilus* HB8 grown in rich medium for 360 min and samples GSM904753-5, obtained from TTHB023-deficient *T. thermophilus* HB8 strains propagated under identical conditions, were analyzed using their NCBI GEO2R program with default settings. Changes in gene expression (LogFC values) and their statistical significance (*p*-values) were determined for each downstream gene within a potentially regulated operon. Likewise, data sets GSM532200-2 (wild-type) and GSM539588-90 in SuperSeries GSE21875 were used to compare gene expression from wild-type and TTHA0167-deficient *T. thermophilus* HB8 strains, to ascertain its involvement in potentially cross-regulated gene expression. Full data from both GEO2R analyses are provided in Supplementary Materials (Tables S2 and S3).

## Figures and Tables

**Table 1:** TTHB023-DNA binding parameters.

Name	Sequence	*k*_on_ (M^−1^·s^−1^)	*k*_off_ (s^−1^)	K_D_ (M)	R^2^
wt	CTGACCGATCGGTCAG	2.739 × 10^5^	5.746 × 10^−4^	2.097 × 10^−9^	0.9830
m1	gTGACCGATCGGTCAG	2.350 × 10^5^	1.072 × 10^−4^	4.560 × 10^−9^	0.9715
m2	CgGACCGATCGGTCAG	1.462 × 10^5^	3.366 × 10^−3^	2.302 × 10^−8^	0.9890
m3	CTcACCGATCGGTCAG	5.491 × 10^5^	3.632 × 10^−3^	6.615 × 10^−9^	0.9891
m4	CTGcCCGATCGGTCAG	1.357 × 10^5^	3.767 × 10^−3^	2.776 × 10^−8^	0.9866
m5	CTGAtCGATCGGTCAG	1.293 × 10^5^	6.868 × 10^−3^	5.311 × 10^−8^	0.9893
m6	CTGACgGATCGGTCAG	6.160 × 10^4^	1.718 × 10^−2^	2.790 × 10^−7^	0.9954
m7	CTGACCcATCGGTCAG	6.734 × 10^4^	1.378 × 10^−2^	2.046 × 10^−7^	0.9928
m8	CTGACCGcTCGGTCAG	9.259 × 10^4^	5.509 × 10^−3^	5.950 × 10^−8^	0.9638
control	cgtcatagaattcg	9.185 × 10^1^	1.272 × 10^−2^	1.385 × 10^−4^	0.9399

(Sequence) Lowercase nucleotides indicate mutation from the consensus TTHB023 sequence.

**Table 2:** TTHB023 consensus sequence sites within the *T. thermophilus* HB8 genome.

Start	End	*p*-Value	*Q*-Value	Sequence	Location	Int?	Gene	Operon
1,254,810	1,254,825	1.25 × 10^−7^	0.199	ATGACCGGTCAGTCAG	−16	Y	*TTHA1315*	S
1,254,810	1,254,825	1.25 × 10^−7^	0.199	CTGACTGACCGGTCAT	−37	Y	*TTHA1316*	S
1,284,538	1,284,553	1.88 × 10^−7^	0.199	ATGACCGGTCGGTCAG	+1	N	*TTHA1342*	3/5
931,257	931,272	4.08 × 10^−7^	0.288	CCGACCGTTCGGTAAG	−10	Y	*TTHA0987*	S
717,396	717,411	7.88 × 10^−7^	0.417	CTTACCGACCGGTTGG	−18	N	*TTHA0750*	5/5
931,253	931,268	2.18 × 10^−6^	0.777	ACTACCGACCGTTCGG	−14	Y	*TTHA0987*	S
13,899	13,914	2.20 × 10^−6^	0.777	TTTACCGACCGGTTGG	−153	Y	*TTHB023*	1/10
13,753	13,768	2.59 × 10^−6^	0.783	CCTACCGACCGGTCGG	−7	Y	*TTHB023*	1/10
1,218,865	1,218,880	4.81 × 10^−6^	1	CCTACCGACCGTTCAA	+1922	N	*TTHA1277*	2/9
1,526,717	1,526,732	5.10 × 10^−6^	1	ATGACCGGTCAGTATT	−44	N	*TTHA1605*	S
1,526,717	1,526,732	5.10 × 10^−6^	1	AATACTGACCGGTCAT	+28	N	*TTHA1606*	1/4

(*p*-value) The probability of a random sequence of the same length matching that position of the sequence with an as good or better score. (*Q*-value) False discovery rate if the occurrence is accepted as significant. (Location) Location of the TTHB023-binding site relative to the start site of translation. (Int?) The TTHB023-binding site is located in an intergenic region. (Operon) Gene position within the postulated operon. (S) No operon, single transcriptional unit.

**Table 3:** TTHB023-promoter binding parameters.

Gene	Sequence	*k*_on_ (M^−1^ s^−1^)	*k*_off_ (s^−1^)	K_D_ (M)	R^2^
*TTHA1315/16*	ATGACCGGTCAGTCAG	2.136 × 10^5^	1.573 × 10^−3^	7.362 × 10^−9^	0.9659
*TTHA0987*(−10)	CCGACCGTTCGGTAAG	2.196 × 10^5^	1.025 × 10^−3^	4.666 × 10^−9^	0.9344
*TTHA0750*	CTTACCGACCGGTTGG	3.972 × 10^5^	1.610 × 10^−4^	4.053 × 10^−10^	0.9678
*TTHA0987*(−14)	ACTACCGACCGTTCGG	2.554 × 10^5^	1.648 × 10^−4^	6.453 × 10^−10^	0.9641
*TTHB023*(−153)	TTTACCGACCGGTTGG	2.723 × 10^5^	2.680 × 10^−4^	9.844 × 10^−10^	0.9586
*TTHB023*(−7)	CCTACCGACCGGTCGG	2.679 × 10^5^	8.443 × 10^−5^	3.151 × 10^−10^	0.9573
*TTHA1605/06*	ATGACCGGTCAGTATT	2.541 × 10^4^	2.124 × 10^−3^	8.357 × 10^−7^	0.9968

(*TTHA1315/16*) A common TTHB023 binding site shared by two bidirectional promoters. (−10) Location of the TTHB023-binding site relative to the start site of translation.

**Table 4:** GEO2R analysis of genes with identified TTHB023-binding sites in their promoters.

Gene	LogFC	Adj. *p*-Value	*p*-Value	t	B
***TTHB023***	−8.51	4.52 × 10^−5^	2.61 × 10^−8^	−33.90	8.80
***TTHA0750***	1.95	0.0199	1.08 × 10^−4^	8.62	1.90
***TTHA0987***	1.83	0.0174	8.97 × 10^−5^	8.91	2.09
*TTHA1315*	−0.0298	0.923	0.792	−0.276	−7.03
*TTHA1316*	0.105	0.781	0.535	0.656	−6.83

(Gene) Bold genes were among the 20 results with the lowest adjusted *p*-values. (LogFC) Log2-fold change between data obtained from TTHB023-deficient and wild-type *T. thermophilus* HB8 strains. (Adj. *p*-value) The *p*-value obtained following multiple testing corrections using the default Benjamini and Hochberg false discovery rate method [15]. (*p*-value) Raw *p*-value. (t) Moderated t-statistic. (B) Log-odds that the gene is differentially expressed.

**Table 5:** TTHB023 potential regulated genes.

Promoter	Operon	Gene	Role	LogFC	Adj *p*-Value
Y	1	*TTHB023*	TetR family transcriptional regulator	−8.51	4.52 × 10^−5^
	2	*TTHB022*	putative acyl-CoA dehydrogenase	0.917	0.145
	3	*TTHB021*	hypothetical protein	1.03	0.118
	4	*TTHB020*	3-oxoacyl-[acyl carrier protein] reductase	1.10	0.170
	5	*TTHB019*	MaoC-related acyl dehydratase	1.46	0.151
	6	*TTHB018*	hypothetical protein	1.15	0.132
	7	*TTHB017*	medium-chain acyl-CoA ligase-related protein	1.05	0.163
	8	*TTHB016*	gluconate 5-dehydrogenase	1.18	0.0712
	9	*TTHB015*	putative acyl-CoA dehydrogenase	1.68	0.0199
	10	*TTHB014*	phosphotransferase	1.86	0.0209
Y	5	*TTHA0750*	3-oxoacyl-ACP reductase	1.95	0.0199
Y	S	*TTHA0987*	beta-ketoadipyl CoA thiolase	1.83	0.0174
Y	S	*TTHA1315*	integral membrane efflux protein	−0.0298	0.923
Y	S	*TTHA1316*	hypothetical protein	0.105	0.781

(1) Number indicates gene position within an operon. (S) No operon, single transcriptional unit. (LogFC) Log2-fold change in expression, TTHB023-deficient:wt *T. thermophilus* HB8 strains, data from GEO [13]. (Adj. *p*-value) The *p*-value obtained following multiple testing corrections using the default Benjamini and Hochberg false discovery rate method [15].

**Table 6:** TTHA0167-promoter binding parameters.

Gene	Sequence	*k*_on_ (M^−1^ s^−1^)	*k*_off_ (s^−1^)	K_D_ (M)	R^2^
*TTHA1315/16*	TGACCGGTCAGTCA	1.237 × 10^5^	1.455 × 10^−4^	1.177 × 10^−9^	0.9758
*TTHA1605/06*	TGACCGGTCAGTAT	1.050 × 10^5^	1.978 × 10^−4^	1.883 × 10^−9^	0.9831

(*TTHA1315/16*) A common TTHA0167 binding site shared by two bidirectional promoters.

## Supplementary Materials

The following are available online at https://www.mdpi.com/2218-273X/10/1/94/s1, Table S1: Oligonucleotides, Table S2: GEO2R analysis of expression profiles from TTHB023-deficient and wild-type *T. thermophilus* HB8 strains, Table S3: GEO2R analysis of expression profiles from TTHA0167-deficient and wild-type *T. thermophilus* HB8 strains, Figure S1: Expression and purification of the recombinant TTHB023 protein, Data S1: REPSA Round 6 fastq sequences, and Data S2: REPSA Round 6 Sequencing1-processed sequences, can be found at https://www.mdpi.com/2218-273X/10/1/94/s1.

## Abbreviations

BLI	Biolayer interferometry
EMSA	Electrophoretic mobility shift assay
FIMO	Find individual motif occurrences
GEO	Gene expression omnibus repository
IISRE	Type IIS restriction endonuclease
MEME	Multiple Em for motif elicitation
REPSA	Restriction endonuclease protection, selection, and amplification
TetR	Tetracycline repressor protein
