# Supplementary material for: General and Genomic DNA-Binding Specificity for the Thermus thermophilus HB8 Transcription Factor TTHB023
Source: Biomolecules. 2020 Jan 6;10(1):94. doi: 10.3390/biom10010094 (PMC7022988; doi:10.3390/biom10010094)
Supplement: Supplementary file 1 [file biomolecules-10-00094-s001.zip › Figure S1.docx]

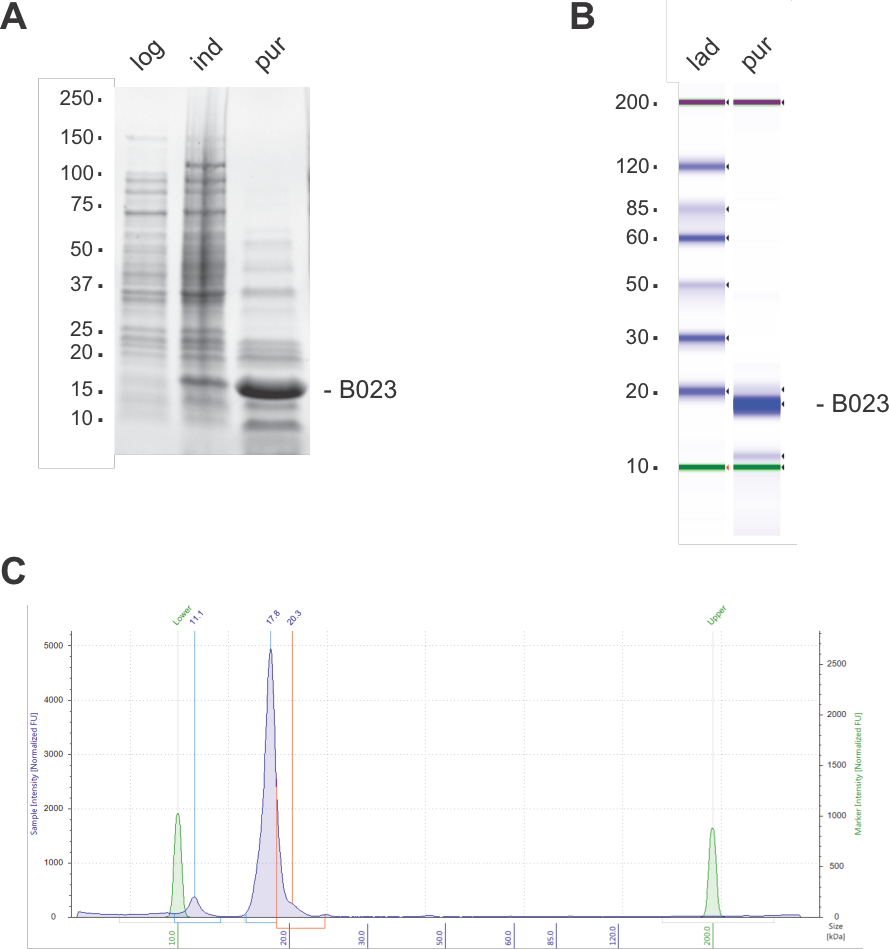


**Figure S1.** Expression and purification of recombinant TTHB023 protein. (**A**) Shown is a Bio-Rad 4-20% SDS-PAGE TGX Stain-Free gradient gel onto which was loaded whole-cell extracts or partially purified fractions containing TTHB023 protein. Lanes shown left to right: (log) 10 µg whole-cell extract from logarithmic growth bacteria, (ind) 26 µg whole-cell extract from bacteria following IPTG-induction for five hours, (pur) 18 μg purified TTHB023 protein. The location of molecular weight standards is indicated at the left of the figure. (**B**) Gel representation of TapeStation P200 ScreenTape data. Lanes shown left to right: (lad) TapeStation P200 ladder, (pur) 0.2 μg purified TTHB023 protein. (**C**) Electropherogram of TapeStation P200 TTHB023 data.
