# Supplementary material for: General and Genomic DNA-Binding Specificity for the Thermus thermophilus HB8 Transcription Factor TTHB023
Source: Biomolecules. 2020 Jan 6;10(1):94. doi: 10.3390/biom10010094 (PMC7022988; doi:10.3390/biom10010094)
Supplement: Supplementary file 1 [file biomolecules-10-00094-s001.zip › Table S1.docx]

**Table 1. Oligonucleotides.**

| Name | Sequence | Length | Purif. | Use |
| --- | --- | --- | --- | --- |
| ST2R24 | CTAGGAATTCGTGCAGAGGTGAATNNNNNNNNNNNNNNNNNNNNNNNNTTACCATCCCTCCAGAAGCTTGGAC | 73 | PAGE | REPSA selection template precursor |
| ST2L | CTAGGAATTCGTGCAGAGGTGAAT | 24 | Desalt | PCR primer |
| IRD7_ST2R | /5IRD700/GTCCAAGCTTCTGGAGGGATGGTAA | 25 | HPLC | 5′ IRDye 700-modified PCR primer |
| A_BC03_ST2R | CCATCTCATCCCTGCGTGTCTCCGACTCAGAAGAGGATTCGATGTCCAAGCTTCTGGAGGGATG | 64 | PAGE | Fusion PCR primer |
| trP1_ST2L | CCTCTCTATGGGCAGTCGGTGATCTAGGAATTCGTGCAGAGGTGA | 45 | PAGE | Fusion PCR primer |
| A_uni | CCATCTCATCCCTGCGTG | 18 | Desalt | PCR primer |
| trP1_uni | CCTCTCTATGGGCAGTCGG | 19 | Desalt | PCR primer |
| REPSAis | CTAGGAATTCGTGCAGAGGTGAATCGTCATAGAATTCGTTACCATCCCTCCAGAAGCTTGGAC | 63 | PAGE | REPSAis control DNA precursor |
| Bio_ST2R | /5BiodT/GTCCAAGCTTCTGGAGGGATG | 22 | HPLC | 5′ biotin-modified PCR primer |
| ST2_B023_R6_wt | AGGAATTCGTGCAGAGGTGAATCTGACCGATCGGTCAGTTACCATCCCTCCAGAAGCTTG | 60 | Desalt | TTHB023 consensus DNA probe precursor |
| ST2_B023_R6_m1 | AGGAATTCGTGCAGAGGTGAATGTGACCGATCGGTCAGTTACCATCCCTCCAGAAGCTTG | 60 | Desalt | TTHB023 mutant 1 DNA probe precursor |
| ST2_B023_R6_m2 | AGGAATTCGTGCAGAGGTGAATCGGACCGATCGGTCAGTTACCATCCCTCCAGAAGCTTG | 60 | Desalt | TTHB023 mutant 2 DNA probe precursor |
| ST2_B023_R6_m3 | AGGAATTCGTGCAGAGGTGAATCTCACCGATCGGTCAGTTACCATCCCTCCAGAAGCTTG | 60 | Desalt | TTHB023 mutant 3 DNA probe precursor |
| ST2_B023_R6_m4 | AGGAATTCGTGCAGAGGTGAATCTGCCCGATCGGTCAGTTACCATCCCTCCAGAAGCTTG | 60 | Desalt | TTHB023 mutant 4 DNA probe precursor |
| ST2_B023_R6_m5 | AGGAATTCGTGCAGAGGTGAATCTGATCGATCGGTCAGTTACCATCCCTCCAGAAGCTTG | 60 | Desalt | TTHB023 mutant 5 DNA probe precursor |
| ST2_B023_R6_m6 | AGGAATTCGTGCAGAGGTGAATCTGACGGATCGGTCAGTTACCATCCCTCCAGAAGCTTG | 60 | Desalt | TTHB023 mutant 6 DNA probe precursor |
| ST2_B023_R6_m7 | AGGAATTCGTGCAGAGGTGAATCTGACCCATCGGTCAGTTACCATCCCTCCAGAAGCTTG | 60 | Desalt | TTHB023 mutant 7 DNA probe precursor |
| ST2_B023_R6_m8 | AGGAATTCGTGCAGAGGTGAATCTGACCGCTCGGTCAGTTACCATCCCTCCAGAAGCTTG | 60 | Desalt | TTHB023 mutant 8 DNA probe precursor |
| ST2_B023_1315/16p | AGGAATTCGTGCAGAGGTGAATATGACCGGTCAGTCAGTTACCATCCCTCCAGAAGCTTG | 60 | Desalt | *TTHA1315/16* promoter DNA probe precursor |
| ST2_B023_0987(–10)p | AGGAATTCGTGCAGAGGTGAATCCGACCGTTCGGTAAGTTACCATCCCTCCAGAAGCTTG | 60 | Desalt | *TTHA0987*(–10) promoter DNA probe precursor |
| ST2_B023_0750p | AGGAATTCGTGCAGAGGTGAATCTTACCGACCGGTTGGTTACCATCCCTCCAGAAGCTTG | 60 | Desalt | *TTHA0750* promoter DNA probe precursor |
| ST2_B023_0987(–14)p | AGGAATTCGTGCAGAGGTGAATACTACCGACCGTTCGGTTACCATCCCTCCAGAAGCTTG | 60 | Desalt | *TTHA0987*(–14) promoter DNA probe precursor |
| ST2_B023_B023(–153)p | AGGAATTCGTGCAGAGGTGAATTTTACCGACCGGTTGGTTACCATCCCTCCAGAAGCTTG | 60 | Desalt | *TTHB023*(–153) promoter DNA probe precursor |
| ST2_B023_B023(–7)p | AGGAATTCGTGCAGAGGTGAATCCTACCGACCGGTCGGTTACCATCCCTCCAGAAGCTTG | 60 | Desalt | *TTHB023*(–7) promoter DNA probe precursor |
| ST2_B023_1605/06p | AGGAATTCGTGCAGAGGTGAATATGACCGGTCAGTATTTTACCATCCCTCCAGAAGCTTG | 60 | Desalt | *TTHA1605/06* promoter DNA probe precursor |

**Figure S1.** Expression and purification of recombinant TTHB023 protein. (**A**) Shown is a Bio-Rad 4-20% SDS-PAGE TGX Stain-Free gradient gel onto which was loaded whole cell extracts or partially purified fractions containing TTHB023 protein. Lanes shown left to right: (log) 10 µg whole cell extract from logarithmic growth bacteria, (ind) 26 µg whole cell extract from bacteria following IPTG-induction for 5 h, (pur) 18 μg purified TTHB023 protein. The location of molecular weight standards is indicated at the left of the figure. (**B**) Gel representation of TapeStation P200 ScreenTape data. Lanes shown left to right: (lad) TapeStation P200 ladder, (pur) 0.2 μg purified TTHB023 protein. (**C**) Electropherogram of TapeStation P200 TTHB023 data.
